# Supplementary material for: HIVprotI: an integrated web based platform for prediction and design of HIV proteins inhibitors
Source: J Cheminform. 2018 Mar 9;10:12. doi: 10.1186/s13321-018-0266-y (PMC5845081; doi:10.1186/s13321-018-0266-y)
Supplement: Supplementary file 2 — Additional file 2. Source code of HIVProtI web server. [file 13321_2018_266_MOESM2_ESM.zip › HIVprotI_Source-code/hivprotI/weka-3-6-6/doc/weka/classifiers/trees/j48/package-summary.html]

weka.classifiers.trees.j48


---


|  |  |  |
| --- | --- | --- |
| **Overview** \| **Package** \| Class \| **Tree** \| **Deprecated** \| **Index** \| **Help** \| **Weka's home** | |  |
| **PREV PACKAGE**   **NEXT PACKAGE** | **FRAMES**    **NO FRAMES**     **All Classes** |  |


---

## Package weka.classifiers.trees.j48

| **Class Summary** | |
| --- | --- |
| **BinC45ModelSelection** | Class for selecting a C4.5-like binary (!) split for a given dataset. |
| **BinC45Split** | Class implementing a binary C4.5-like split on an attribute. |
| **C45ModelSelection** | Class for selecting a C4.5-type split for a given dataset. |
| **C45PruneableClassifierTree** | Class for handling a tree structure that can be pruned using C4.5 procedures. |
| **C45PruneableClassifierTreeG** | Class for handling a tree structure that can be pruned using C4.5 procedures and have nodes grafted on. |
| **C45Split** | Class implementing a C4.5-type split on an attribute. |
| **ClassifierSplitModel** | Abstract class for classification models that can be used recursively to split the data. |
| **ClassifierTree** | Class for handling a tree structure used for classification. |
| **Distribution** | Class for handling a distribution of class values. |
| **EntropyBasedSplitCrit** | "Abstract" class for computing splitting criteria based on the entropy of a class distribution. |
| **EntropySplitCrit** | Class for computing the entropy for a given distribution. |
| **GainRatioSplitCrit** | Class for computing the gain ratio for a given distribution. |
| **GraftSplit** | Class implementing a split for nodes added to a tree during grafting. |
| **InfoGainSplitCrit** | Class for computing the information gain for a given distribution. |
| **ModelSelection** | Abstract class for model selection criteria. |
| **NBTreeClassifierTree** | Class for handling a naive bayes tree structure used for classification. |
| **NBTreeModelSelection** | Class for selecting a NB tree split. |
| **NBTreeNoSplit** | Class implementing a "no-split"-split (leaf node) for naive bayes trees. |
| **NBTreeSplit** | Class implementing a NBTree split on an attribute. |
| **NoSplit** | Class implementing a "no-split"-split. |
| **PruneableClassifierTree** | Class for handling a tree structure that can be pruned using a pruning set. |
| **SplitCriterion** | Abstract class for computing splitting criteria with respect to distributions of class values. |
| **Stats** | Class implementing a statistical routine needed by J48 to compute its error estimate. |

---


|  |  |  |
| --- | --- | --- |
| **Overview** \| **Package** \| Class \| **Tree** \| **Deprecated** \| **Index** \| **Help** \| **Weka's home** | |  |
| **PREV PACKAGE**   **NEXT PACKAGE** | **FRAMES**    **NO FRAMES**     **All Classes** |  |


---
